# Estimation of the demand for palliative care in non-oncologic patients in Chile

**DOI:** 10.1186/s12904-022-01122-z

**Published:** 2023-01-12

**Authors:** Nicolás Armijo, Tomás Abbot, Manuel Espinoza, Ximena Neculhueque, Carlos Balmaceda

**Affiliations:** 1 Health Technology Assessment Unit, Clinical Research Center, School of Medicine, Pontificia Universidad Católica de Chile, Santiago, Chile; 2 Department of Public Health, Faculty of Medicine, Health Technology Assessment Unit, Clinical Research Center, School of Medicine, Pontificia Universidad Católica de Chile, Pontificia Universidad Católica de Chile, Diagonal Paraguay, 362 Santiago, Chile; 3 Ministry of Health, Santiago, Chile; 4 Centre for Health Economics, University of York, York, UK

**Keywords:** Palliative care, Mortality, Health services needs and demand, Chronic disease, Comorbidity, Forecasting

## Abstract

**Background:**

Access to palliative care is an emerging global public health challenge. In Chile, a palliative care law was recently enacted to extend palliative care coverage to the non-oncologic population. Thus, a reliable and legitimate estimate of the demand for palliative care is needed for proper health policy planning.

**Objective:**

To estimate the demand for Palliative Care in Chile.

**Methodology:**

Diseases likely to require palliative care were identified according to literature and expert judgement. Annual deaths of diseases identified were estimated for the periods 2018–2020. Demand estimation corresponds to the identification of the proportion of deceased patients requiring palliative care based on the burden of severe health-related suffering. Finally, patient-years were estimated based on the expected survival adjustment.

**Results:**

The estimated demand for palliative care varies between 25,650 and 21,679 patients depending on the approximation used. In terms of annual demand, this varies between 1,442 and 10,964 patient-years. The estimated need has a minor variation between 2018 and 2019 of 0.85% on average, while 2020 shows a slightly higher decrease (7.26%).

**Conclusion:**

This is a replicable method for estimating the demand of palliative care in other jurisdictions. Future studies could approach the demand based on the decedent population and living one for a more precise estimation and better-informed health planning. It is hoped that our methodological approach will serve as an input for implementing the palliative care law in Chile, and as an example of estimating the demand for palliative care in other jurisdictions.

**Supplementary Information:**

The online version contains supplementary material available at 10.1186/s12904-022-01122-z.

## Background

Palliative care (PC) is the active total care of patients whose disease does not respond to curative treatment. PC aims to achieve the best possible quality of life for patients and their families [1]. World Health Organization (WHO) emphasizes that PC is a component of universal health coverage, integrated into the Sustainable Development Goals. [2–4].

PC is not only designed for patients with end-stage cancer but also for people suffering from non-oncological diseases from stages before the so-called end-of-life stage [5]. PC is focused on those patients who experience severe health-related suffering (SHS), that is when the suffering produced by an injury or illness cannot be alleviated without the intervention of a professional and when it compromises physical, social, spiritual, and/or emotional functioning [6]. Thus, people suffering from a disease with SHS can benefit from PC, which not only contemplate therapies focused on managing the pathology, but also those actions and research-oriented to understand and manage ─ more efficiently ─ the distressing clinical complications suffered by these patients [7].

Access to PC is an emerging challenge for global public health. Patients with diseases that require end-of-life PC experience a significant economic burden [8]. Several studies have reported that the costs related to end-of-life care represent around 25–30% of insurer medical expenditures [9–11]. In addition, there is a relevant cost component related to patient care that is incurred by society. [12–14]. Thus, access to PC in the coverage schemes should be prioritized because the evidence supports that this health service alleviates the suffering of patients, families, and saves money for healthcare systems and society [15–17]. Moreover, annually more than 61 million people worldwide experience about 6 billion days of SHS that can be potentially alleviated with access to a PC [18]. Regarding the need for PC, it is estimated that by 2060, 47% of worldwide deaths will experience a high SHS burden, potentially requiring PC [19].

Chile has made progress in providing access to PC since the implementation of the Explicit Health Guarantees (GES) regime, which included access to a set of PC services for cancer patients [20]. Although this is an important achievement, there is still a need to promote interdisciplinary management, provide continuous care with good quality home assistance and extend its coverage to non-oncologic diseases. [21].

According to the Global Atlas of Palliative Care report, around 30% of the population requiring PC are patients with malignant neoplasms [9]. In the Chilean settings, the latter reveals a major gap in terms of access to PC, with a significant fraction of the target population (around 70% of patients who experience SHS) excluded from the social security system. Regarding the distributional consequences of the lack of access, this is more detrimental to patients with low socioeconomic status, since they tend to have greater barriers to access to health care, and therefore, to PC [22]. In addition, the demand for non-oncological PC may be increasing by demographic and epidemiological changes that result in a high incidence of complications of chronic diseases. [9, 23, 24].

However, in the context of a new palliative care law which was recently launched in Chile [25], PC services will be expanded to patients with non-oncologic diseases. For this purpose, the local authority required a precise estimate of the resources needed to provide them to the healthcare sector adequately. Although some efforts have been made to develop estimates of the population in need of PC [6, 7, 19, 26–32]; none of them has reached the technical robustness and legitimacy to support the national health policy. This report is the result of the collaboration between the Ministry of Health of Chile (MoH) and the technical group of Health Technology Assessment at Pontificia Universidad Católica de Chile, to generate a validated methodology and a reliable estimate of the demand for PC in Chile.

### Methodology

To estimate the need for PC, we considered the aim of the law project proposed by the Chilean Ministry of health, which is to provide PC coverage in the last year of life through domiciliary care. Thus, a retrospective approach was used based on annual death records. This approach assumes that the number of people needing palliative care during the year represents those who finally died during the same period. This approach is considered adequate in those cases where palliative care services are focused on providing end-of-life care [6, 28, 31], which is precisely the purpose of the present study.

The estimation of the need for PC was carried out according to the following steps: (1) Identification of the diseases susceptible to receiving non-oncological PC; (2) Estimation of the annual deaths whose cause corresponds to the group of diseases identified; (3) Estimation of the proportion of deceased patients for each disease that will require palliative care; (4) Estimation of the number of patient-years based on the expected survival adjustment.

### Identification of diseases susceptible to receiving non-oncologic palliative care

There are several sources related to the identification of diseases susceptible to receiving PC. Three different approaches were considered to explore those variations for the identification of diseases susceptible to non-oncologic PC. For the first approach, a group of diseases defined according to their ICD-10 category (International Classification of Diseases, 10th edition) were identified based on the proposal of Murtagh et al.[28] and the Lancet Commission report [6], which included a larger number of diseases. In addition, we validated this list with local clinical experts (a physician specializing in PC with more than ten years of clinical expertise, academic training in this field and advisor experience to the Health Ministry in PC policies), which led to the incorporation some additional codes. Oncologic pathologies were discarded, but the code for specific neoplasms associated with HIV.

For the second approach, we restricted the set of diseases identified in the first approach excluding those, according to expert judgment, who have a low probability of utilization of PC for end-of-life care. They included HIV-related infectious diseases, acute life-threatening diseases such as acute myocardial infarction, pulmonary embolism, acute pericarditis, acute myocarditis, acute renal failure, or infectious diseases of the respiratory system. The main assumption is that the patient suffering any of these conditions as the cause of death and diagnosed close to the date of death, would not have been a candidate for PC before that date. Finally, for the third approach, we used the group of pathologies proposed by Murtagh et al. [28]. The groups of pathologies to be considered for each approach, together with the ICD-10 codes, are presented in Table 1.

**Table 1:** Health conditions are included in the estimate of the population requiring palliative care

The proposed list of diseases	CD-10 Code	Health condition
(i) The list of diseases proposed by Murtagh et al., considering the groups of diseases analyzed by the Lancet Commission.	B20-B24, F01-F04, G10, G12, G20-G26, G30, G35-G37, G90.3, I00-I52, I60-I69, J06, J09, J10-J18, J20 -J22, J40-J47, J60-J65, J96, K70-K77, N17-N19, N28, R54, M00-M97	Diseases caused by Human Immunodeficiency Virus (HIV), Organic mental disorders, including symptomatic disorders, Extrapyramidal and other movement disorders, other degenerative CNS disorders. Demyelinating CNS conditions; cerebral palsy and other paralytic syndromes, Acute rheumatic fever, chronic rheumatic heart disease, Hypertensive diseases, Ischemic heart disease, Pulmonary heart disease and diseases of the pulmonary circulation, Other forms of heart disease, Cerebrovascular diseases, Chronic lung disease, lung disease due to external agents, interstitial lung disease, other diseases of the respiratory system, Other diseases of the respiratory system, Liver disease, Lung failure, Lung disease due to external agents, Interstitial lung disease, other diseases of the respiratory system, liver disease, renal failure, senility, and musculoskeletal disease.
(ii) The list of diseases proposed by Murtagh et al. contrasted with the groups of diseases analyzed by the Lancet Commission (restricted).	B21, F01-F04, G10, G12, G20-G26, G30, G35-G37, G90.3, I00-I52 (except I21, I24, I26, I30, I40), I60-I69, J40-J47, J60-J65, J96, K70-K77, N18-N19, N28, R54, M00-M97	The following diseases were excluded: Infectious and parasitic diseases resulting from Human Immunodeficiency Virus (HIV), Other specified diseases resulting from Human Immunodeficiency Virus (HIV), Other conditions resulting from Human Immunodeficiency Virus, Unspecified diseases resulting from Human Immunodeficiency Virus (HIV), Acute myocardial infarction, Other acute ischemic heart disease, Pulmonary embolism, Acute pericarditis, Acute myocarditis, Acute upper respiratory infections from multiple sites, and Unspecified sites, Influenza due to identified zoonotic or pandemic influenza viruses, Influenza due to identified seasonal influenza viruses, Influenza, unidentified virus, Viral pneumonia, not elsewhere classified, Pneumonia due to Streptococcus pneumonia, Pneumonia due to Haemophilus influenzae, Bacterial pneumonia, not elsewhere classified, Pneumonia due to other infectious organisms not elsewhere classified, Pneumonia in diseases classified elsewhere, Pneumonia, organism unspecified Acute bronchitis, Acute bronchiolitis, Acute lower respiratory infection unspecified.
(iii) Diseases included in Murtagh et al.	B20-B24, F01, F03, G10, G12.2, G20, G23.1, G30, G35, G90.3, I00-I52, I60-I69, J06-J18, J20-J22, J40-J47, J96, K70-K77, N17, N18, N28, R54.	Diseases caused by Human Immunodeficiency Virus (HIV), Organic mental disorders, Demyelinating diseases of the central nervous system, Parkinson’s disease, Huntington’s disease, Multisystem degeneration, Acute rheumatic fever, chronic rheumatic heart disease, Hypertensive diseases, Ischemic heart disease, Pulmonary heart disease and diseases of the pulmonary circulation, Cerebrovascular diseases, Acute upper respiratory tract infections, Influenza and Pneumonia, Liver diseases, Renal failure, Senility.

### Estimate of annual deaths

Data on the number and causes of death were obtained from the Department of Health Statistics and Information of the MoH which is publicly available [33]. This provides cause of death data for each fatality in Chile and other demographic details. These data were filtered according to the cause of death for 2018–2020 and by month.

### Estimate the proportion of deceased patients for each disease receiving palliative care

To estimate the proportion of deceased patients receiving PC for each disease, we used the weights developed by the Lancet Commission [6]. Briefly, the expert’s commission developed a multiplier that informs the proportion of people from each health condition experiencing SHS [6] and hence who required PC. For those pathologies not considered by the Lancet Commission, similarities were discussed with clinical experts to match each code in order to provide an equal proportion of palliative care needs [6]. Supplementary Table 1 reports the proportion of patients who will require PC for each pathology. We applied this approach to all scenarios, including the one that considers the set of pathologies reported by Murtagh et al.

### Estimation of the number of patient-years based on the expected survival adjustment

The purpose of the present study was to estimate the demand expressed as the number of patient-years. This estimate is equivalent to the expected number of patients who will require PC for a whole year. Standardization is needed because not all patients who die in a year require the same amount of PC time. While some will require a few days, others will require full-year support.

To estimate the average non-oncologic PC length of stay (LOS), similar derivation between oncologic and non-oncologic average PC LOS were assumed. This derivation was obtained from Jordan et al. [34]. This study reported an average oncologic PC LOS of 28 days and 24.27 days for non-oncologic PC LOS. Consequently, 13.3% of reduction was calculated and applied to Chilean data regarding the average oncologic PC. In addition, there is an average of 180 days of survival for cancer patients who receive PC in Chile [35]. Thus, the expected average non-oncologic average PC LOS were 156 days. This approach assumes that the hazard of death between day one and the day end of the month is constant. This value provides information for forecasting deaths and can be applied to allocating resources to palliative care.

## Results

Table 2 presents the total deaths and the population that will effectively require PC according to the diseases susceptible to receiving non-oncologic PC for each one of the proposed approaches over the years 2018–2020. An average variation (considering estimates i, ii, and iii) of 0.85% is observed between the periods 2018–2019; and an average decrease of 7.26% between the periods 2019–2020. It should be noted, that for approaches (i) and (iii), about 55% of the registered deaths would require PC, whereas, for the approach (ii), the proportion is about 61%.

**Table 2:** Annual decreases and estimated population requiring palliative care for the years 2018, 2019, and 2020

	2018		2019		2020		
Approach	Decedents	PC	Decedents	PC	Decedents	PC	Average PC
Estimate (i)	47,323	26,073	48,610	26,525	44,555	24,352	25,650
Estimate (ii)	36,022	22,110	35,953	21,831	34,267	21,095	21,679
Estimate (iii)	46,410	25,501	47,713	25,959	43,742	23,838	25,099

*PC *Palliative care. Estimate (i): Group of diseases proposed by Murtagh et al. and considering the groups of diseases analysed by the Lancet Commission. Estimate (ii): Estimate (i) restricted. Estimate (iii): Group of diseases proposed by Murtagh et al.

Regarding the expected PC LOS for non-oncologic diseases, Table 3 reports the total PC LOS expected by the three estimates. It is shown a minimum of 526,149 days and a maximum of 4,001,950 days. Moreover, it is expected between 1,442 and 10,964 patient-years when standardization is applied.

**Table 3:** Expected non-oncologic palliative care length of stay and patient years

Approach	PC LOS^a^	Patient years^a^	PC LOS^b^	Patient years^b^
Estimate (i)	622,526	1,706	4,001,950	10,964
Estimate (ii)	526,149	1,442	3,382,389	9,267
Estimate (iii)	609,153	1,669	3,915,982	10,729

^a^Jordan et al. non-oncologic PC LOS was used for the estimation. ^b^non-oncologic PC LOS was estimate assuming same derivation between oncologic and non-oncologic LOS of Jordan et al., and then applied to available Chilean data

## Discussion

The present study estimated the expected number of patients who need PC in one year in Chile, evaluating different scenarios. Estimates range from 12,825 to 10,839 patient-years, with a minor variation between 2018 and 2019 of 0.85% on average, and a more significant decrease in 2020 (7.26%). The variation in the need for PC in the 2020 period could be explained by the fact that the groups of diseases considered in our estimate could be an aggravating factor for the mortality of COVID-19 [36], with this disease being the main cause of death.

This is the first estimate of the need for PC for non-oncology patients in Chile and South America. However, other studies have reported some estimates worldwide [19] [7], England [1, 37], Scotland [38], Australia [29], Germany [30], Italy [39], and Malaysia [31]. Nevertheless, different estimates used alternative methodologies and a different health conditions. In this context, alternative approaches like the one reported in this manuscript may add value because it proposes a new set of conditions that achieved consensus and legitimacy to support the demand estimates.

Some of the differences between our approach and others refer to the consideration of oncologic diseases. As mentioned above, in Chile PC for cancer is provided through a different coverage scheme (20). Regarding non-oncologic PC, through the weights revealed by the Lancet Commission, which inform the fraction of individuals who will require PC by illness, based on SHS, our approach could be considered more refined in terms of demand estimation [6, 9]. Most studies assumed that all patients who will die from PC-susceptible disease will receive such care, because this does not hold for all patients, an overestimation of the actual demand will occur [27, 28, 37].

Although Etkind et al. attempt to address this issue by introducing pain prevalence in their estimates, the results of the present work can be understood as more accurate because the use of the SHS encompasses the requirement for PC in more dimensions than pain, and because we considered a more individualized PC requirement weight. In contrast, Etkind et al. considered an expected pain prevalence by disease groups (organ failure, dementia, and others) that might ignore within-group heterogeneity [1].

Our estimation also considered the PC LOS needed. This information is relevant as it allows us to make a more precise estimate of the expected annual demand. Thus, the range of total expected PC LOS was estimated between 1,442 and 10,964 patient year depending on the set of assumptions made.

The results obtained allow us to identify the first challenges of implementing PC at the primary health care level. This is of utmost importance as primary health care has an essential role to play in the fight against health inequities. Integrating non-oncological PC into primary health care could help to reduce these inequalities, something that specialist palliative care teams have not been able to achieve [40].

Our estimates have some limitations, the data are collected according to death registries, which may be underreported. This may occur with diseases such as dementia or Parkinson’s disease, which lack of registry may impact underestimating the need for PC [28, 41–43]. Also, as our estimate is based on the diagnosis of the first cause of death, the need for PC may be underestimated because of comorbidities that could increase its demand [19, 44, 45]. Furthermore, although we used the weights validated by both the Lancet Commission expert panel and our experts (for those conditions not included in the Lancet Commission), they are not precise estimates and should be validated in future research [19]. Additionally, our non-oncologic PC LOS estimation lacks local reliable data. The assumptions made may not reflect the average non-oncologic PC LOS despite the discussion with clinical expert. Therefore, new registers are needed in order to make more accurate estimate.

Other methods of estimating PC demand have been reported [6, 32, 46, 47]. The Lancet Commission develops a robust estimate based on the deceased considering that population in their last year of life, and of the non-deceased, considering PC requirements before the last year of life [6, 9]. To undertake the above approximation, prevalence data are required for all disease groups considered in the methodology, which is a major challenge for future research. In addition, given that the estimate of the present study is within the context of a future law on non-oncological PCs, this estimate is plausible since the law considers, in the first place, PCs in the last six months of life.

Estimating the demand for PC is a key resource for the planning of health policies that take care of the lack of access to PC. Coverage of diseases requiring PC care needs to increase over time, as health policies for non-oncological PC provision have the potential to alleviate the suffering of a large proportion of patients and their families. It is hoped that this estimate will allow accounting for the relevance of addressing this public health problem to organize patient-centred PC provision, improve coordination of healthcare, and thus strengthen the public health system by ensuring equity in access to PC.

## Supplementary Information

**Additional file 1: Supplementary Table 1. **The estimated proportion of decedents requiringpalliative care (PC).

## Data Availability

This paper reports an analysis of publicly available summary data on mortality. The datasets supporting the conclusions of this article is available in the Department of Health Statistics and Information of the Ministry of Health of Chile repository as follow: Mortality data: https://deis.minsal.cl/#datosabiertos Accessed June 2021.

## Abbreviations

PC: Palliative Care
WHO: World Health Organization
SHS: Health-related suffering
GES: Explicit Health Guarantees
MoH: Ministry of Health of Chile
ICD-10: International Classification of Diseases 10th edition
