# Supplementary material for: Estimation of the demand for palliative care in non-oncologic patients in Chile
Source: BMC Palliat Care. 2023 Jan 12;22:5. doi: 10.1186/s12904-022-01122-z (PMC9834031; doi:10.1186/s12904-022-01122-z)
Supplement: Supplementary file 1 — Additional file 1: Supplementary Table 1. The estimated proportion of decedents requiringpalliative care (PC). [file 12904_2022_1122_MOESM1_ESM.docx]

Supplementary Table 1. The estimated proportion of decedents requiring palliative care (PC).

| ICD section | Health condition (ICD code) | The proportion of decedents requiring PC. |
| --- | --- | --- |
| B20 | HIV disease resulting in mycobacterial infection | 100% |
| B21 | HIV disease resulting in malignant neoplasms | 100% |
| B22 | HIV disease resulting in other specified diseases | 100% |
| B23 | HIV disease resulting in other conditions | 100% |
| B24 | Unspecified HIV disease | 100% |
| F01 | Vascular dementia | 80% |
| F02 | Dementia in other diseases classified elsewhere | 80% |
| F03 | Unspecified dementia | 80% |
| F04 | Organic amnesic syndrome, not induced by alcohol and other psychoactive substances | 80% |
| G10 | Huntington disease | 65% |
| G12 | Spinal muscular atrophy and related syndromes | 65% |
| G20 | Parkinson disease | 65% |
| G21 | Secondary parkinsonism | 65% |
| G22 | Parkinsonism in diseases classified elsewhere | 65% |
| G23 | Other degenerative diseases of basal ganglia | 65% |
| G24 | Dystonia | 65% |
| G25 | Other extrapyramidal and movement disorders | 65% |
| G26 | Extrapyramidal and movement disorders in diseases classified elsewhere | 65% |
| G30 | Alzheimer disease | 65% |
| G35 | Multiple sclerosis | 100% |
| G37 | Other demyelinating diseases of central nervous system | 65% |
| G90.3 | Multi-system degeneration of the autonomic nervous system | 65% |
| I00 | Rheumatic fever without mention of heart involvement | 65% |
| I01 | Rheumatic fever with heart involvement | 65% |
| I02 | Rheumatic chorea | 65% |
| I05 | Rheumatic mitral valve diseases | 65% |
| I06 | Rheumatic aortic valve diseases | 65% |
| I07 | Rheumatic tricuspid valve diseases | 65% |
| I08 | Multiple valve diseases | 65% |
| I09 | Other rheumatic heart diseases | 65% |
| I10 | Essential (primary) hypertension | 70% |
| I11 | Hypertensive heart disease | 70% |
| I12 | Hypertensive renal disease | 70% |
| I13 | Hypertensive heart and renal disease | 70% |
| I15 | Secondary hypertension | 70% |
| I20 | Angina pectoris | 5% |
| I21 | Acute myocardial infarction | 5% |
| I22 | Subsequent myocardial infarction | 5% |
| I23 | Certain current complications following acute myocardial infarction | 5% |
| I24 | Other acute ischaemic heart diseases | 5% |
| I25 | Chronic ischaemic heart disease | 5% |
| I26 | Pulmonary embolism | 5% |
| I27 | Other pulmonary heart diseases | 5% |
| I28 | Other diseases of pulmonary vessels | 5% |
| I30 | Acute pericarditis | 40% |
| I31 | Other diseases of pericardium | 40% |
| I32 | Pericarditis in diseases classified elsewhere | 40% |
| I33 | Acute and subacute endocarditis | 40% |
| I34 | Nonrheumatic mitral valve disorders | 40% |
| I35 | Nonrheumatic aortic valve disorders | 40% |
| I36 | Nonrheumatic tricuspid valve disorders | 40% |
| I37 | Pulmonary valve disorders | 40% |
| I38 | Endocarditis, valve unspecified | 40% |
| I39 | Endocarditis and heart valve disorders in diseases classified elsewhere | 40% |
| I40 | Acute myocarditis | 40% |
| I41 | Myocarditis in diseases classified elsewhere | 40% |
| I42 | Cardiomyopathy | 40% |
| I43 | Cardiomyopathy in diseases classified elsewhere | 40% |
| I44 | Atrioventricular and left bundle-branch block | 40% |
| I45 | Other conduction disorders | 40% |
| I46 | Cardiac arrest | 40% |
| I47 | Paroxysmal tachycardia | 40% |
| I48 | Atrial fibrillation and flutter | 40% |
| I49 | Other cardiac arrhythmias | 40% |
| I50 | Heart failure | 40% |
| I51 | Complications and ill-defined descriptions of heart disease | 40% |
| I52 | Other heart disorders in diseases classified elsewhere | 40% |
| I60 | Subarachnoid haemorrhage | 65% |
| I61 | Intracerebral haemorrhage | 65% |
| I62 | Other nontraumatic intracranial haemorrhage | 65% |
| I63 | Cerebral infarction | 65% |
| I64 | Stroke, not specified as haemorrhage or infarction | 65% |
| I65 | Occlusion and stenosis of precerebral arteries, not resulting in cerebral infarction | 65% |
| I66 | Occlusion and stenosis of cerebral arteries, not resulting in cerebral infarction | 65% |
| I67 | Other cerebrovascular diseases | 65% |
| I68 | Cerebrovascular disorders in diseases classified elsewhere | 65% |
| I69 | Sequelae of cerebrovascular disease | 65% |
| J06 | Acute nasopharyngitis | 50% |
| J09 | Influenza due to identified zoonotic or pandemic influenza virus | 50% |
| J10 | Influenza due to identified seasonal influenza virus | 50% |
| J11 | Influenza, virus not identified | 50% |
| J12 | Viral pneumonia, not elsewhere classified | 50% |
| J13 | Pneumonia due to Streptococcus pneumoniae | 50% |
| J14 | Pneumonia due to Haemophilus influenzae | 50% |
| J15 | Bacterial pneumonia, not elsewhere classified | 50% |
| J16 | Pneumonia due to other infectious organisms, not elsewhere classified | 50% |
| J17 | Pneumonia in diseases classified elsewhere | 50% |
| J18 | Pneumonia, organism unspecified | 50% |
| J20 | Acute bronchitis | 50% |
| J21 | Acute bronchiolitis | 50% |
| J22 | Unspecified acute lower respiratory infection | 50% |
| J40 | Bronchitis, not specified as acute or chronic | 50% |
| J41 | Simple and mucopurulent chronic bronchitis | 80% |
| J42 | Unspecified chronic bronchitis | 80% |
| J43 | Emphysema | 50% |
| J44 | Other chronic obstructive pulmonary disease | 80% |
| J45 | Asthma | 80% |
| J46 | Status asthmaticus | 50% |
| J47 | Bronchiectasis | 50% |
| J60 | Coalworker pneumoconiosis | 50% |
| J61 | Pneumoconiosis due to asbestos and other mineral fibres | 50% |
| J62 | Pneumoconiosis due to dust containing silica | 50% |
| J63 | Pneumoconiosis due to other inorganic dusts | 50% |
| J64 | Unspecified pneumoconiosis | 50% |
| J65 | Pneumoconiosis associated with tuberculosis | 50% |
| J96 | Respiratory failure, not elsewhere classified | 80% |
| K70 | Alcoholic liver disease | 95% |
| K71 | Toxic liver disease | 95% |
| K72 | Hepatic failure, not elsewhere classified | 95% |
| K73 | Chronic hepatitis, not elsewhere classified | 95% |
| K74 | Fibrosis and cirrhosis of liver | 95% |
| K75 | Other inflammatory liver diseases | 95% |
| K76 | Other diseases of liver | 95% |
| K77 | Liver disorders in diseases classified elsewhere | 95% |
| N17 | Acute renal failure | 45% |
| N18 | Chronic kidney disease | 45% |
| N19 | Unspecified kidney failure | 45% |
| N28 | Other disorders of kidney and ureter, not elsewhere classified | 45% |
| R54 | Senility | 65% |
| M00 | Pyogenic arthritis | 70% |
| M01 | Direct infections of joint in infectious and parasitic diseases classified elsewhere | 70% |
| M02 | Reactive arthropathies | 70% |
| M03 | Postinfective and reactive arthropathies in diseases classified elsewhere | 70% |
| M05 | Seropositive rheumatoid arthritis | 70% |
| M06 | Other rheumatoid arthritis | 70% |
| M07 | Psoriatic and enteropathic arthropathies | 70% |
| M08 | Juvenile arthritis | 70% |
| M09 | Juvenile arthritis in diseases classified elsewhere | 70% |
| M10 | Gout | 70% |
| M11 | Other crystal arthropathies | 70% |
| M12 | Other specific arthropathies | 70% |
| M13 | Other arthritis | 70% |
| M14 | Arthropathies in other diseases classified elsewhere | 70% |
| M15 | Polyarthrosis | 70% |
| M16 | Coxarthrosis | 70% |
| M17 | Gonarthrosis | 70% |
| M18 | Arthrosis of first carpometacarpal joint | 70% |
| M19 | Other arthrosis | 70% |
| M20 | Acquired deformities of fingers and toes | 70% |
| M21 | Other acquired deformities of limbs | 70% |
| M22 | Disorders of patella | 70% |
| M23 | Internal derangement of knee | 70% |
| M24 | Other specific joint derangements | 70% |
| M25 | Other joint disorders, not elsewhere classified | 70% |
| M30 | Polyarteritis nodosa and related conditions | 70% |
| M31 | Other necrotizing vasculopathies | 70% |
| M32 | Systemic lupus erythematosus | 70% |
| M33 | Dermatopolymyositis | 70% |
| M34 | Systemic sclerosis | 70% |
| M35 | Other systemic involvement of connective tissue | 70% |
| M36 | Systemic disorders of connective tissue in diseases classified elsewhere | 70% |
| M40 | Kyphosis and lordosis | 70% |
| M41 | Scoliosis | 70% |
| M42 | Spinal osteochondrosis | 70% |
| M43 | Other deforming dorsopathies | 70% |
| M45 | Ankylosing spondylitis | 70% |
| M46 | Other inflammatory spondylopathies | 70% |
| M47 | Spondylosis | 70% |
| M48 | Other spondylopathies | 70% |
| M49 | Spondylopathies in diseases classified elsewhere | 70% |
| M50 | Cervical disc disorders | 70% |
| M51 | Other intervertebral disc disorders | 70% |
| M53 | Other dorsopathies, not elsewhere classified | 70% |
| M54 | Dorsalgia | 70% |
| M60 | Myositis | 70% |
| M61 | Calcification and ossification of muscle | 70% |
| M62 | Other disorders of muscle | 70% |
| M63 | Disorders of muscle in diseases classified elsewhere | 70% |
| M65 | Synovitis and tenosynovitis | 70% |
| M66 | Spontaneous rupture of synovium and tendon | 70% |
| M67 | Other disorders of synovium and tendon | 70% |
| M68 | Disorders of synovium and tendon in diseases classified elsewhere | 70% |
| M70 | Soft tissue disorders related to use, overuse and pressure | 70% |
| M71 | Other bursopathies | 70% |
| M72 | Fibroblastic disorders | 70% |
| M73 | Soft tissue disorders in diseases classified elsewhere | 70% |
| M75 | Shoulder lesions | 70% |
| M76 | Enthesopathies of lower limb, excluding foot | 70% |
| M77 | Other enthesopathies | 70% |
| M79 | Other soft tissue disorders, not elsewhere classified | 70% |
| M80 | Osteoporosis with pathological fracture | 70% |
| M81 | Osteoporosis without pathological fracture | 70% |
| M82 | Osteoporosis in diseases classified elsewhere | 70% |
| M83 | Adult osteomalacia | 70% |
| M84 | Disorders of continuity of bone | 70% |
| M85 | Other disorders of bone density and structure | 70% |
| M86 | Osteomyelitis | 70% |
| M87 | Osteonecrosis | 70% |
| M88 | Paget disease of bone | 70% |
| M89 | Other disorders of bone | 70% |
| M90 | Osteopathies in diseases classified elsewhere | 70% |
| M91 | Juvenile osteochondrosis of hip and pelvis | 70% |
| M92 | Other juvenile osteochondrosis | 70% |
| M93 | Other osteochondropathies | 70% |
| M94 | Other disorders of cartilage | 70% |
| M95 | Other acquired deformities of musculoskeletal system and connective tissue | 70% |
| M96 | Postprocedural musculoskeletal disorders, not elsewhere classified | 70% |
| M97 | Biomechanical lesions, not elsewhere classified | 70% |
